# Natural History of Acute Dysentery

**Published:** 1875-08

**Authors:** Austin Flint


Natural History of Acute Dysentery—*Prof. Austin Flint, Sr.* * * * The foregoing ten cases are all which I have observed with reference to the study of the natural history of the disease.

The disease in no instance ended fatally. The duration, dating from the beginning of diarrhœa to the time when convalescence was declared, varied from 6 to 31 days. In one case only was the duration 21 days, and in this case diarrhœa had existed for 14 days before the occurrence of the first dysenteric dejection, the duration, in this case, after the latter dejection had occurred, being 7 days; throwing out this case, the next longest duration was 14 days. The shortest duration was 6 days. The mean duration of all the other cases is 11 1-5 days. The mean duration of nine cases, excluding the case in which diarrhœa preceded the dysenteric evacuations for fourteen days is 10½ days.

These results of the analyses serve, so far as inferences from the small number of cases are warrantable, 1st, to corroborate the conclusion drawn from the study of cases in which treatment more or less active had been employed, in respect of fatality, namely, that sporadic dysentery, in a temperate climate, tends intrinsically to recovery; 2d, to show that this disease has a self-limited duration. The number of cases analyzed, few as they are, perhaps suffice to establish these two important points in the natural history of the disease.

With reference to the inquiry in how far treatment may affect the duration, it is of interest to compare the foregoing results of of the analysis with those of my analysis of 49 cases in 1853. The mean duration in thirty of the latter cases, ending in recovery, as already stated, was 9 5-6 days; this is a little less than the mean duration of the cases now analyzed. The difference is small, and it may be inferred from the comparison that in the 30 cases analyzed in 1853, the treatment had very little, if any, effect in abridging the duration of the disease.

The 10 cases now analyzed were all hospital cases, and, perhaps, the comparison were fairer if limited to the hospital cases among those formerly analyzed. Of the 30 cases, 13 were in hospital practice. The mean duration in these 13 cases, as already stated, was just 13 days, which is somewhat longer than the mean duration in the cases now analyzed. If we were to draw an inference from this latter comparison, in respect of the effect of treatment, it would be that the duration was thereby somewhat lengthened. This would probably be a strained inference, and I shall content myself with the conclusion that the duration, in the cases formerly, as well as those now, analyzed, was determined by the self-limitation of the disease.

The duration in 9 of the 10 cases, from the date of the beginning of diarrhœa to the date of the discharge from the hospital, varied from 6 to 39 days, the mean duration being 19 8-9 days. The stay of patients in hospital after convalesence does not necessarily denote unusual tardiness in recuperation, whereas a speedy discharge is generally evidence of rapidity in the restoration of health. The duration to the date of discharge, in these cases, is chiefly of interest with regard to the latter point. In 3 of the cases the date of discharge was the same as the date of the convalescence; these patients were considered as, at once, in a condition to leave the hospital. But, comparing the mean duration, in all the cases, to convalescence, with the mean duration to the date of discharge, the latter exceeds the former by only a fraction over 8 days. The analysis, therefore, in this respect, sustains a conclusion drawn from my former analytical study of this disease, namely, that convalescence is usually rapid.

In no case was there a relapse after convalescence during the stay in hospital. In some instances a feculent diarrhœa followed the dysenteric dejections, but the latter, having once ceased, did not recur in any case. It should be noted in this connection, that the patients were allowed to take such solid food as they desired, as soon as they had a desire for it, the previous diet consisting chiefly of milk. The diarrhœa following the dysenteric dejections may have been due to indigestion, and, if so, the presence of undigested aliment in the large intestine did not reproduce the dysentery. The analysis in this point of view sustains a conclusion drawn from my former analysis, namely, the disease is not one which tends to relapse, resembling in this respect typhus and typhoid fever, pneumonia, etc. This fact is to be added to the self-limited duration, and other facts proving the specific character of the affection.

In no case did a chronic follow the acute affection, as is not infrequently observed in tropical climates. As regards a result of my former analysis, to which reference has already been made, namely, the diminished susceptibility to the cause or causes of dysentery after the disease has been once experienced, this group of cases furnishes no facts of importance. Nothing is known of the patients after their discharge, excepting in one case; Peter

G., case number 6, was lately in hospital with alcoholism, and he stated that he had not had dysentery since he left the hospital. It may be mentioned that the previous history in all the cases (which I did not introduce in my abstracts) was more or less full, and in no instance had the patient previously had dysentery.

The duration from the first dysenteric dejection to the date of convalescence was noted in 8 cases. The longest duration was 12 days, the shortest 5 days, and the mean duration was 8½ days. Comparing the latter with the mean duration from the beginning of diarrhœa to convalescence in all the cases (11 1-5 days), the difference, a fraction over 3 days, shows the mean duration of the diarrhœa preceding the dysenteric characteristics.

In the 10 cases now studied, as in the 49 cases previously analyzed, the disease finished its course without any important complication or secondary affection. In none of the cases which I have collected was the disease associated with articular rheumatism or paraplegia, affections to which dysentery has been supposed sometimes to give rise.

To recapitulate the practical conclusions drawn from the study of ten hospital cases of sporadic dysentery, with reference to the natural history of the disease—

1st. The disease in a temperate latitude tends, without treatment, to recovery.

2d. It is a self-limited disease, and its duration is but little, if at all, abridged by methods of treatment now and heretofore in vogue.

3d. Convalescence is as rapid when active measures of treatment have not been employed, as in cases actively treated.

4th. Relapses do not occur in the cases in which the disease has been allowed to pursue its own course without active treatment.

5th. Sporadic dysentery, in a temperate climate, does not eventuate in a chronic form of the disease; in other words, it does not lead to ulceration or other lesions of the mucous membrane of the large intestine, and it does not involve any tendency to complications or secondary affections.—*Am. Jour. Med. Sci.*

